# Cadmium Disrupts Subcellular Organelles, Including Chloroplasts, Resulting in Melatonin Induction in Plants

**DOI:** 10.3390/molecules22101791

**Published:** 2017-10-23

**Authors:** Hyoung-Yool Lee, Kyoungwhan Back

**Affiliations:** Department of Biotechnology, Bioenergy Research Center, College of Agriculture and Life Sciences, Chonnam National University, 77 Yongbong-ro, Buk-gu, Gwangju 61186, Korea; xanthine@naver.com

**Keywords:** aluminum, cadmium, chloroplasts, rice, tobacco

## Abstract

Cadmium is a well-known elicitor of melatonin synthesis in plants, including rice. However, the mechanisms by which cadmium induces melatonin induction remain elusive. To investigate whether cadmium influences physical integrities in subcellular organelles, we treated tobacco leaves with either CdCl_2_ or AlCl_3_ and monitored the structures of subcellular organelles—such as chloroplasts, mitochondria, and the endoplasmic reticulum (ER)—using confocal microscopic analysis. Unlike AlCl_3_ treatment, CdCl_2_ (0.5 mM) treatment significantly disrupted chloroplasts, mitochondria, and ER. In theory, the disruption of chloroplasts enabled chloroplast-expressed serotonin *N*-acetyltransferase (SNAT) to encounter serotonin in the cytoplasm, leading to the synthesis of *N*-acetylserotonin followed by melatonin synthesis. In fact, the disruption of chloroplasts by cadmium, not by aluminum, gave rise to a huge induction of melatonin in rice leaves, which suggests that cadmium-treated chloroplast disruption plays an important role in inducing melatonin in plants by removing physical barriers, such as chloroplast double membranes, allowing SNAT to gain access to the serotonin substrate enriched in the cytoplasm.

## 1. Introduction

Melatonin (*N*-acetyl-5-methoxytryptamine) is a well-known bioactive molecule found in all living organisms tested so far. It functions as a neurohormone in animals [1,2,3] and as a biostimulator in plants [4,5,6]. Its universal distribution is associated with its endosymbiotic origins from the alpha-protobacteria and cyanobacteria that evolved into mitochondria and chloroplasts in eukaryotes [7]. In particular, plants respond to various environmental stresses—including drought [8,9], salt [10,11], cold [12,13,14], heavy metals [15,16,17,18], high temperature [19,20], and pathogens [21,22,23,24]—by inducing melatonin synthesis to confer resistance against these stimuli. During the past decades, many abiotic stresses—such as light [25,26,27], hydrogen peroxide [28], and cold [29]—were applied to plants to induce melatonin biosynthesis, among which cadmium was the most efficient elicitor of melatonin induction. For example, in the detached leaves of rice, the highest and second highest levels of melatonin were induced by cadmium treatment and peroxydizing herbicide butafenacil, respectively, whereas lead failed to induce melatonin [30]. Cadmium also triggered increased melatonin in other plants—including tomato, alfalfa, and Arabidopsis [16,17,31]—which suggests beneficial effects of melatonin against cadmium stress. This hypothesis has been proven, as many melatonin-rich plants exhibit resistance against cadmium stress [6,16]. 

Melatonin synthesis requires four enzymatic steps: first, tryptophan is decarboxylated into tryptamine by tryptophan decarboxylase (TDC), and then serotonin is synthesized via tryptamine 5-hydroxylase (T5H). Next is catalysis by serotonin *N*-acetyltransferase (SNAT). Finally, *N*-acetylserotonin *O*-methyltransferase (ASMT) catalyzes the final reaction [32]. Cadmium and nitric oxide (NO) treatments equally induced all melatonin biosynthetic gene transcripts except SNAT, but an increase in melatonin occurred only as a result of cadmium treatment [33]. This result indicates that the ample production of serotonin by NO treatment was not coupled with melatonin synthesis because of possible inaccessibility of serotonin into chloroplasts in which the enzyme SNAT is constitutively expressed. Thus, we hypothesized that cadmium treatment may disrupt chloroplast structures that trigger SNAT to gain access to and easily encounter serotonin for melatonin production. 

To prove this hypothesis, we treated tobacco leaves with both cadmium and aluminum and examined them using confocal microscopic analysis to see whether the structures of subcellular organelles—such as chloroplasts, mitochondria, and ER—were affected. We found that cadmium disrupted chloroplasts, mitochondria, and ER structures, whereas aluminum (Al) only disrupted ER slightly. In addition, Al treatment failed to induce melatonin in rice seedlings, whereas Cd did induce melatonin. The reason for using rice plant species to measure melatonin was its easy and ample induction of melatonin upon Cd compared to tobacco. In fact, tobacco leaves marginally induced melatonin in response to Cd as did tomato [16]. These results suggest that chloroplast disruption due to cadmium plays an important role in inducing melatonin synthesis in plants. 

## 2. Results and Discussion

### 2.1. Cadmium Treatment Disrupts the Structures of Chloroplasts, Mitochondria, and ER

We used 0.5 mM cadmium to investigate whether cadmium treatment affects the structures of various subcellular organelles, such as chloroplasts, mitochondria, and ER, as this concentration induced the highest level of melatonin in rice [33]. Aluminum was used for the control treatment because it increased melatonin in soybean [15]. Visualization of these organelle structures was detected by chlorophyll fluorescence, MitoTracker Green FM staining, and HDEL-CFP (cyan fluorescent protein) marker protein. 

**Figure 1:**
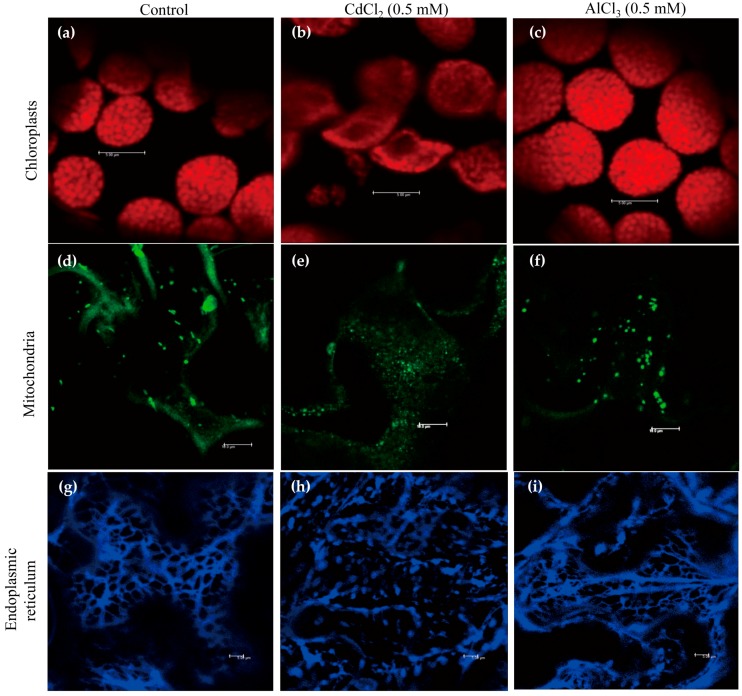
Effects of cadmium and aluminum on the structures of subcellular organelles as shown by confocal microscopy. (**a**–**c**) Structures of chloroplasts: (**a**) Control; (**b**) Cadmium treatment; (**c**) Aluminum treatment; (**d**–**f**) Structures of mitochondria: (**d**) Control; (**e**) Cadmium treatment; (**f**) Aluminum treatment; (**g**–**i**) Structures of ER: (**g**) Control; (**h**) Cadmium treatment; (**i**) Aluminum treatment. Tobacco leaves were infiltrated with heavy metals (0.5 mM) for 4 h and subjected to confocal microscopic analysis. Bars = 10 µm (chloroplasts and mitochondria), 5 µm (ER).

In response to cadmium treatment, chloroplasts were swollen, distorted, and worn down, whereas control and aluminum treatments maintained the integrity of the chloroplasts (Figure 1a–c). Similarly, cadmium treatment abolished the structures of the mitochondria, but no such damage was observed in mitochondria treated with aluminum, which suggests the susceptibility of mitochondria to cadmium (Figure 1d–f). By contrast, cadmium and aluminum treatments ruined a tubular ER network, whereas the control showed a normal ER network (Figure 1g–i). Of the three subcellular organelles, ER seemed to be the most sensitive to heavy metals because ER structures were labile to aluminum treatment.

These results clearly show that cadmium treatment severely disrupts the integrity of chloroplasts, mitochondria, and ER. Such disruption could result in the leaking of endogenous SNAT enzymes [9,32] out of these organelles’ double membranes, where they could readily encounter cytoplasmic serotonin for *N*-acetylserotonin synthesis followed by the production of melatonin via the catalytic reaction of cytoplasmic ASMT enzyme in response to cadmium stress. Unlike cadmium, aluminum treatment had no effect on the wreckage of chloroplasts and mitochondria except ER, indicative of an ineffective elicitor for melatonin induction. ER also plays an important role in serotonin synthesis rather than melatonin synthesis because T5H is a P450 enzyme localized in ER responsible for serotonin synthesis. The effects of ER disruption in view of melatonin induction by cadmium remain to be examined, but it seems unlikely that ER damage is the major cause of melatonin induction by cadmium, because aluminum also destroyed the ER structure. 

### 2.2. Melatonin Induction in Rice Seedlings Following Cadmium and Aluminum Treatments

**Figure 2:**
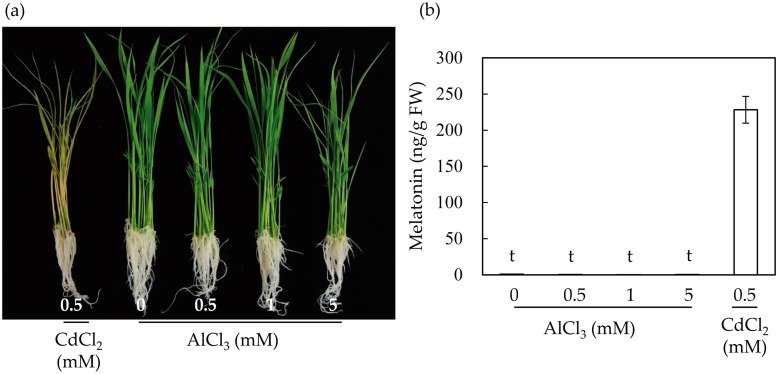
Induction of melatonin in response to cadmium and aluminum treatments in seven-day-old rice seedlings. (**a**) Phenotypes of rice seedlings upon cadmium and aluminum treatments; (**b**) Quantification of melatonin by HPLC. Data represent mean ± standard error of the mean, *n* = 3. Rice seedlings grown for seven days were challenged with cadmium (0.5 mM) or aluminum of varying concentrations for three days under continuous light. FW, fresh weight; t denotes 1 ng/g FW.

To further determine the effects of aluminum on the induction of melatonin in rice plants, we treated seven-day-old rice seedlings with varying levels of aluminum for three days. As a control, rice seedlings were treated with 0.5 mM cadmium for three days. The rice seedlings treated with cadmium showed phenotypes with severely necrotizing leaves (Figure 2a). By contrast, rice seedlings treated with 0.5–1 mM aluminum exhibited relatively healthy phenotypes, but rice seedlings treated with 5 mM aluminum showed necrosis in some parts of the leaves. Next, we determined the melatonin levels in the rice seedlings treated with cadmium and aluminum. As shown in Figure 2b, melatonin induction was not observed in the rice seedlings treated with aluminum, even at a concentration of 5 mM aluminum, whereas treatment with cadmium (0.5 mM) produced 228 ng/g fresh weight (FW). Together, these results suggest that in light of the subcellular organelles and SNAT enzyme catalysis, one of the main reasons for melatonin induction was the disruption of chloroplasts upon treatment with cadmium.

## 3. Materials and Method

### 3.1. Confocal Microscopic Analysis

Tobacco (*Nicotiana benthamiana*) plants were grown at 25 °C under a 16 h light/8 h dark period in an environmentally controlled growth room. The pBIN61-HDEL-CFP plasmid as the ER fluorescence marker was kindly donated by Dr. HG Kang (Texas State University, San Marcos, TX, USA). This plasmid was transformed to the *Agrobacterium tumefaciens* strain GV2260 using the freeze–thaw method, and transient expression analyses were performed as described by Voinnet et al. [34].

Two-week-old *N. benthamiana* leaves were syringe-infiltrated with GV2260 carrying the pBIN61-HDEL-CFP plasmid, then incubated in the growth room for two days. These tobacco leaves were further treated with cadmium (0.5 mM) or aluminum (0.5 mM) for 4 h, and images were analyzed using a Leica TCS-SP5 confocal microscope (×63 oil immersion objective) (Leica, Wetzlar, Germany). CFP was excited with the Mercury arc lamp (435 nm) and emitted light was collected between 480 and 520 nm. Images were processed using Leica LAS-AF Version 1.8.2 (Leica, Wetzlar, Germany). 

MitoTracker Green FM probes, 50 µg (Invitrogen, Carlsbad, CA, USA), were sequentially dissolved with DMSO to obtain 50 mM stock solution and diluted with 3 mM MES solution (pH5.6) to obtain a working concentration of 1 µM. Following treatment with cadmium (0.5 mM) or aluminum (0.5 mM) for 4 h, tobacco leaves were stained with the MitoTracker probe for 15 min at room temperature and rinsed with fresh water before confocal analysis. The probe was excited with a blue argon ion laser (488 nm), and emitted light was collected between 494 and 546 nm.

The chloroplast autofluorescence was excited with a blue argon laser (488 nm), and emitted light was collected from 660 to 731 nm. 

### 3.2. Rice Seedlings

Rice seeds (*Oryza sativa* cv. Dongjin) were surface-sterilized and germinated on half-strength Murashige and Skoog (MS) medium (MB Cell, Seoul, Korea) in vertically oriented polystyrene square dishes (SPL Life Science, Pocheon-si, Korea). The environmental conditions for rice seedlings’ growth were 28 °C under a 12 h light/12 h dark cycle at a photosynthetic photon flux density of 150 µmol m^−2^ s^−1^. Seven days after seeding, the seedlings were harvested for cadmium and aluminum treatments. The rice variety cv. Dongin (japonica type) was not Al tolerant and Cd sensitive. It was reported that all japonica types of rice were equally sensitive to Al stress [35].

### 3.3. Cadmium and Aluminum Treatments and Melatonin Quantification in Rice Seedlings

Seven-day-old seedlings were transferred to 50 mL polypropylene conical tubes containing 25 mL water with 0.5 mM cadmium (CdCl_2_) or varying concentrations of aluminum (AlCl_3_). These seedlings were incubated for three days under continuous light as described previously [32]. The rice samples (shoot parts) were frozen in liquid nitrogen and pulverized to a powder using a TissueLyser II instrument (Qiagen, Tokyo, Japan). The powder (100 mg) was extracted with 1.5 mL chloroform for 1 h at room temperature. The chloroform extracts were evaporated until dry and dissolved in 0.1 mL 42% methanol. Aliquots of 10 µL were subjected to HPLC with a fluorescence detector system (Waters, Milford, MA, USA), as described previously [32].

## 4. Conclusions

In summary, cadmium treatment induced melatonin synthesis in rice plants, whereas treatment with aluminum did not induce melatonin synthesis. In terms of the structures of subcellular organelles, cadmium treatment led to the wreckage of chloroplasts and mitochondria, whereas aluminum treatment did not break down either chloroplasts or mitochondria. The resulting disruption of chloroplasts by cadmium facilitates the encounter of substrate serotonin with chloroplastidic SNAT enzymes, which in turn results in a huge induction of melatonin in rice plants. Our results confirm that one of the main reasons for the failure of melatonin induction despite the huge increase in serotonin upon NO treatment [33] or senescence [5] is interference with the entry of serotonin into chloroplasts in which SNAT enzymes reside. On the other hand, the cadmium-induced melatonin synthesis via the disruption of chloroplasts in cytoplasm should be interpreted with caution because the CoA is also very important for melatonin synthesis and there is high level of CoA in chloroplast relative over in the cytoplasm. Additionally, it is worth noting that the physicochemical properties of CdCl_2_ and AlCl_3_ may play different roles in melatonin synthesis as well. For example, AlCl_3_ is a potent Lewis acid, whilst CdCl_2_ is a mild Lewis acid. Moreover, AlCl_3_·H_2_O liberates HCl whereas CdCl_2_ forms complex ions, such as [CdCl_4_]^2−^. Further studies in this regard are therefore needed in the near future in view of melatonin induction in plants.
